# Bedtime and the Budget: Longitudinal, Actor–Partner Connections between Sleep Quality and Financial Management Behaviors in Newlywed Couples

**DOI:** 10.3390/ijerph20010055

**Published:** 2022-12-21

**Authors:** Matthew T. Saxey, Jeffrey P. Dew, Jeremy B. Yorgason

**Affiliations:** School of Family Life, Brigham Young University, Provo, UT 84602, USA

**Keywords:** financial behaviors, financial well-being, marital satisfaction, sleep quality

## Abstract

Research from the American Psychological Association suggests that approximately 67% of U.S. adults are getting more or less sleep than desired, and over 80% of U.S. adults ages 18–43 are stressed about finances. Cross-sectional evidence suggests that there may be a connection between the two. That is, some cross-sectional research suggests a contemporaneous association between sleep quality and finances. Using two waves of newlywed dyadic data (*N* = 1497 couples), we estimated a longitudinal structural equation model to test actor–partner associations between husbands’ and wives’ sleep quality and financial management behaviors. In these associations, we examined husbands’ and wives’ marital satisfaction as potential mediating variables. We found that both husbands’ and wives’ sleep quality longitudinally predicted their own and their partner’s financial management behaviors. Additionally, husbands’ and wives’ sleep quality—through wives’ marital satisfaction—indirectly and longitudinally predicted wives’ financial management behaviors. As financial practitioners encourage newlywed couples to consistently experience quality sleep, their financial management behaviors may benefit. We suggest that for newlywed couples, both partners’ bedtime may be longitudinally connected to both partners’ management of their budget.

## 1. Introduction

Many Americans are stressed about their finances. For example, research conducted by the American Psychological Association around the start of the COVID-19 pandemic (2020) found that money was the source of stress for 64% of United States (U.S.) adults [1]. More recent estimates (2022) suggest that, among U.S. adults ages 18–43, over 80% report being stressed about money [2]. Although scholars have examined many predictors of financial management behaviors—which might help mitigate financial stress [3], even during times of economic hardship [4]—few have examined how biosocial issues, such as sleep quality, might play a role in predicting financial management behaviors. Examining sleep quality’s impact on financial management behaviors especially matters today because roughly two in three Americans report sleeping more or less than desired [5]. In this study, we examined longitudinal associations between sleep quality and financial management behaviors in newlywed couples. Examining these connections among newlywed couples matters because these couples often struggle with finances [6,7,8]. That is, many newlywed couples might be included in the over 80% of U.S. adults ages 18–43 who are stressed about finances [2,9].

Two recent studies suggest that sleep quality is associated with individuals’ financial matters, specifically financial management behaviors and financial exploitation of the elderly [10,11]. We build upon these important studies’ findings in three ways. First, both of the previous studies utilized cross-sectional data. We used longitudinal data, which can suggest whether sleep at one time point is related to later financial management behaviors (i.e., financial behaviors that help individuals meet financial goals and achieve financial well-being) [12]. Understanding sleep quality’s efficacy as a longitudinal predictor of financial management behaviors may inform interventions of financial practitioners (i.e., financial planners, counselors, educators, and clinicians) who help newlywed couples struggling with finances. Another way that we build on prior research is by using data from both spouses in the marriage (i.e., dyadic data). Dyadic data might provide insights into whether one spouse’s sleep quality might benefit the other spouse’s financial management behaviors.

Third, neither of the previous studies tested potential mediating variables between sleep quality and financial management behaviors. That is, we still do not understand those factors that might link sleep quality and financial management behaviors. In our study, we tested marital satisfaction as a potential mediating variable between sleep quality and financial management behaviors. Understanding mediating variables between sleep quality and financial management behaviors may inform practice by suggesting additional avenues for intervention in connections between sleep quality and financial management behaviors. Testing whether marital satisfaction links sleep quality and sound financial management behavior could also benefit researchers who study these topics.

### 1.1. Theoretical Support for Sleep Quality Predicting Financial Management Behaviors

The association between sleep quality and financial management behaviors can be understood through the five-factor model of personality (FFMP) [13]. The FFMP is a widely used model that identifies the importance of considering how personality traits such as openness, extraversion, agreeableness, neuroticism, and conscientiousness might play a role in predicting behavior. Scholars recently theorized that conscientiousness (i.e., exemplifying qualities such as self-discipline, dutifulness, and orderliness) [13] about health behaviors, such as sleep, might be positively associated with conscientiousness about finances—specifically, conscientiousness with financial management behaviors [10]. Indeed, sleep quality positively predicted positive financial management behaviors [10]. Conceptualizing sleep quality as an indicator of conscientiousness related to health behaviors is supported by recent work that suggests sleep quality and conscientiousness are positively associated [14,15].

Put simply, greater sleep conscientiousness might make it easier to display conscientiousness with finances. For example, a recent meta-analysis revealed that conscientiousness is positively associated with adaptive emotion regulation strategies and negatively associated with maladaptive emotion regulation strategies [13]. Better sleep quality has also been linked to better emotion regulation [16]. Thus, having better emotion regulation strategies from greater sleep conscientiousness might make it easier to manage one’s finances better. That is, experiencing higher quality sleep—evidencing conscientiousness—and having better emotion regulation strategies might make it easier to, for example, withstand impulse purchases [17] and exhibit intentionality in financial management behaviors [18]. Emotional regulation and financial management are often interconnected [18], so having better control of one’s emotions—from higher quality sleep—might engender better control of one’s finances. 

Despite theoretical support for a connection between sleep quality and financial management behaviors, scholars have scarcely examined this possibility. As we searched the literature involving both sleep quality and financial management behaviors, we found only two studies that tested sleep quality’s impact on financial management of some kind. One study of individuals ages 18–65 and older (*N* = 8128) found that getting at least seven hours of sleep at night was significantly associated with positive financial management behaviors [10]. A study of financial exploitation of the elderly found that adults ages 50 or older who reported experiencing financial exploitation also reported experiencing problems with sleep [11]. These findings provide a beginning to understanding sleep quality’s association with financial management behaviors, and we sought to further understand this association through examining a potential mediating variable.

### 1.2. Marital Satisfaction as a Mediating Variable

Marital satisfaction might help explain the association between sleep quality and financial management behaviors. Indeed, conscientiousness with sleep might also improve the likelihood that one is conscientiousness in his/her marriage. Based on a review of the literature, scholars have theorized that getting more quality sleep might improve psychological and physiological functioning, and improved psychological and physiological functioning might improve marital functioning (e.g., the quality of a marriage) [19]. Recent research supports this theory by demonstrating positive associations between sleep quality and marital quality [20,21,22]. For example, a study of older adults found that relationship quality and sleep quality were positively linked [20]. Further research conducted with older married adults showed a positive longitudinal link between marital quality and sleep quality [21]. Other research with newlywed couples also suggests a positive connection between sleep quality and marital satisfaction [22].

Marital satisfaction might be a mediating variable because it also predicts financial management behaviors [23]. Although a majority of couple finance scholars view marital satisfaction, or similar variables, as outcomes rather than predictors [24], recent longitudinal evidence suggests that marital satisfaction could be a stronger predictor of financial management behaviors rather than vice versa [23]. Dew et al. (2021) theorized that those who have higher satisfaction with their marriage might be more likely to invest in the marriage by putting in the effort necessary to manage finances well, which was supported by their findings [23]. Therefore, it could be that higher marital satisfaction motivates more conscientious financial management behaviors [23].

This perspective was alluded to by Skogrand et al. (2011) in their seminal study about the financial management practices of couples with self-proclaimed great marriages [25]. Suggesting that financial management behaviors alone, the authors argued, might promote a quality marriage might be unrealistic [25]. That is, couples might first need to be conscientious in their marriage (e.g., develop trust) so that they can subsequently be conscientious in a difficult area for couples to navigate [8,26], such as their finances (e.g., develop trust with finances). This line of thinking is also supported by other research that suggests that couples who are nearing divorce tend to spend down their assets [27], suggesting that a lack of conscientiousness within a marriage might predict a lack of conscientiousness with finances.

Because sleep quality predicts marital satisfaction and marital satisfaction predicts financial management behaviors, marital satisfaction might link sleep quality and financial management behaviors. For example, as supported by previous theorizing [19], consistently getting quality sleep may help spouses have more self-control [28]. With more self-control from higher-quality sleep, spouses may be more likely to be conscientiousness in their marriage and report higher marital satisfaction [21,22]. A better marriage, therefore, may provide more motivation than a lower-quality marriage to conscientiously manage finances [23,25,27]. Accordingly, we tested the following hypotheses:

**Hypothesis** **1.** *Sleep quality will predict positive financial management behaviors over time for both husbands and wives.*

**Hypothesis** **2.** *The association between sleep quality and positive financial management behaviors will be indirect and marital satisfaction will be the linking variable.*

### 1.3. Actor–Partner Effects

Within the context of a marital relationship, Troxel et al. suggest considering the dyadic nature of sleep [19], which we sought to do through the use of the actor–partner interdependence model [29]. Actor effects occur when one’s own levels of an explanatory variable (e.g., one’s sleep quality) are associated with levels of one’s own dependent variables (e.g., one’s personal financial management behaviors). Partner effects occur when one’s own levels of an explanatory variable (e.g., one’s sleep quality) are associated with one’s partner’s dependent variables (e.g., one’s spouse’s financial management behaviors) [29]. Because spouses’ sleep quality is likely interdependent [30], one’s spouse’s quality of sleep might have actor and partner effects when predicting marital satisfaction. 

Evidence suggests that one spouse’s sleep problems are associated with the other spouse’s poorer marital happiness [31]. Perhaps a spouse’s poor sleep quality might lead to poorer self-control [28], and this poorer self-control might make it more difficult for this spouse to be conscientiousness in how they treat their spouse, which might have implications for the poorly treated spouse’s marital satisfaction. Specific to our study, the poorly treated spouse’s marital satisfaction might then lessen, which might make it less motivating for this spouse to conscientiously manage finances [23].

We suspected the possibility of actor–partner effects in connections between sleep quality and financial management behaviors also based on the previous literature about sleep concordance (i.e., couples’ synchrony in sleep) and relationship functioning [32,33]. For example, husbands’ and wives’ sleep concordance has been found to be connected with both husbands’ and wives’ marital satisfaction [32]. This finding suggests that the way both partners experience sleep might have implications for both partners’ marital satisfaction. This marital satisfaction, whether high or low, might then predict financial management behaviors [23]. Therefore, following theory [19] and the previous literature, we hypothesized actor and partner effects:

**Hypothesis** **3.** *Husbands and wives’ sleep quality will predict positive financial management behaviors through marital satisfaction via both actor and partner effects.*

## 2. Materials and Methods

### 2.1. Data and Sample

Data for the current study came from waves two (T2) and three (T3) of the *Couple Relationships and Transition Experiences* (CREATE) study [34]. The CREATE data set is a nationally representative sample of newlywed couples in the U.S. that was obtained through a two-stage cluster stratification sample design. After receiving Institutional Review Board (IRB) approval, U.S. counties were randomly selected based on county population size; marriage, divorce, and poverty rates; and the racial–ethnic distribution. Then publicly available marriage records were obtained and used to invite couples to participate. To be included in the study, at least one partner in the dyad must have been between the ages of 18 and 36 at the start of the study, at least one partner in the dyad must have been in a first marriage, and the couple must have been living in the U.S. Of the 11,889 couples who were invited to participate, 2181 couples confirmed interest and met each of the inclusion criteria. In total, 90% of the participants were married in 2014, 6% were married in 2015, and 4% were married in 2013. 

We used criteria based on our hypotheses to draw specific participants from the CREATE data. First, because we were interested in testing actor–partner effects, participants had to have their spouse participate in the survey. Second, because we lacked statistical power to be able to analyze group differences between same-sex and different-sex marriages, we removed the few same-sex couples from the data. Finally, couples must have participated in the first three waves of the CREATE survey (and remain married during that time). The final analytical sample included 1497 couples. For demographic information for the 1497 couples, see Table 1. For more information on the CREATE study, see James et al. [34]. 

### 2.2. Measures

#### 2.2.1. Sleep Quality

Sleep quality was measured by using 19 items from the Pittsburg Sleep Quality Index (PSQI) [35]. The items we used from the PSQI are grouped together to make up seven subscales that measure sleep quality, sleep duration, sleep latency, sleep medications, habitual sleep efficiency, sleep disturbances, and daytime dysfunction. Scores from the subscales were aggregated to generate an overall sleep quality score, which ranged from 0 to 21, with higher scores measuring greater sleep dysfunction. We reverse-coded PSQI scores so that higher scores represented higher-quality sleep (i.e., for ease of interpretability). Reliability estimates of the PSQI for husbands (α = 0.63) and wives (α = 0.63) at T2 suggest that subscales are fairly reliable—yet they also assess multiple dimensions of sleep. 

We recognize that the Cronbach’s alphas are below the typically accepted level of 0.7. However, Cronbach’s alphas are usually used to evaluate whether items in a unidimensional scale measure the same concept. The PSQI was designed to measure multiple dimensions of sleep, as described above. Therefore, because the PSQI was not designed to be unidimensional, we were not surprised that the Cronbach’s alphas were below 0.7. In addition to its good psychometric properties [35], the PSQI has also been test–retest validated [36]. For these reasons, we felt justified in using this widely used measure of sleep quality.

#### 2.2.2. Marital Satisfaction

We used four items that were based on measures from the Couples Satisfaction Index [37]. For the first two items, partners were asked, “In general, how satisfied are you with your relationship?” and “How rewarding is your relationship with your partner?” In response, participants rated their experience on a scale of 0 (not at all) to 5 (completely). For the third item, partners were shown the statement, “I have a warm and comfortable relationship with my partner.” Each participant rated his/her experience on a scale of 0 (not at all true) to 5 (completely true). For the last item, partners were shown the statement, “Please select the answer that describes the degree of happiness, all things considered, of your relationship.” Based on their experiences, participants responded on a scale of 0 (extremely unhappy) to 6 (perfect). Higher scores represent greater marital satisfaction. In our analytical sample, considerable reliability was achieved for T2 marital satisfaction (α = 0.94 for both wives and husbands).

#### 2.2.3. Financial Management Behaviors

We used four items to measure financial management behaviors that were drawn from the Financial Management Behavior Scale [38]. Participants were asked to gauge how often (in the last six months) they engaged in behaviors such as “Paid all your bills on time” and “Saved money from every paycheck.” In response to the statements such as these, participants rated their experiences on a scale of 1 (never) to 5 (always). In the response options, partners could also select −1 (does not apply); we treated responses of −1 as a 1 (never), because “does not apply” means that the participants did not engage in those behaviors. For T3 financial management behaviors in our analytical sample, adequate reliability was achieved (α = 0.75 for husbands; α = 0.74 for wives).

#### 2.2.4. Control Variables

We adjusted the analyses for a few control variables. These control variables included husbands’ and wives’ age, education, and race/ethnicity at wave one (T1) and annual household income at T2. Age was measured in years since birth. Education was measured on a scale of 1 (less than high school) to 7 (advanced degree JD, PhD, PsyD, etc.). We controlled for race/ethnicity by including three race control variables for both husbands and wives (i.e., totaling six control variables for race/ethnicity). Specifically, we included whether or not husbands and wives were Black (coded as 0 = Other, and 1 = Black), Hispanic (coded as 0 = Other, and 1 = Hispanic), or Other (coded as 0 = Not Other, and 1 = Other). White non-Hispanic was the omitted category. Annual household income was measured on a scale of 1 ($0–9999) to 16 ($150,000 or more).

### 2.3. Data Analysis

**Figure 1:**
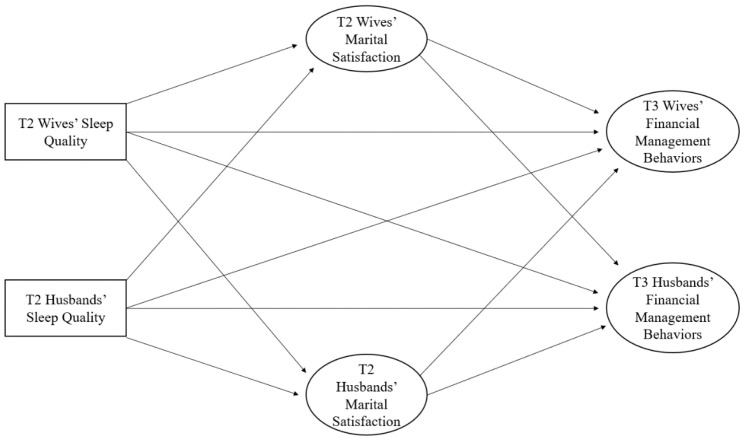
Conceptual model of longitudinal actor–partner connections between sleep quality and financial management behaviors. Note: Rectangles represent observed variables, and ellipses represent latent variables. For concision, we do not show controlling for husbands’ and wives’ age, education, race, and annual household income.

We tested our hypotheses by using a single structural equation model (SEM; see Figure 1) in Mplus (version 8.4). We estimated direct associations between husbands’ and wives’ T2 sleep quality and T2 marital satisfaction, T2 marital satisfaction and T3 financial management behaviors, and T2 sleep quality and T3 financial management behaviors. We also estimated indirect associations between husbands’ and wives’ T2 sleep quality and husbands’ and wives’ T3 financial management behaviors through husbands’ and wives’ T2 marital satisfaction. To estimate the statistical significance of indirect effects, we examined 95% confidence intervals with 5000 bootstraps to ensure that indirect effects were statistically different from zero [39]. In the model, we included all control variables previously mentioned. To account for non-independence in the dyadic nature of the data, we correlated husbands’ and wives’ reports of sleep quality, marital satisfaction, and financial management behaviors.

We adopted certain standards to judge the relative strength of our findings. Specifically, to assess the effect sizes of the standardized direct associations, we used Cohen’s (1988) recommended cutoffs: approximately 0.1 to 0.3 is considered small, 0.3 to 0.5 is considered medium, and 0.5 and higher is considered large [40]. To identify the effect sizes of the standardized indirect associations, we used Kenny’s (2021) cutoffs: roughly 0.01 to 0.09 is considered small, 0.09 to 0.25 is considered medium, and 0.25 and higher is considered large [41]. To identify the effect sizes of the explained variance of constructs (i.e., *R*^2^), we used Cohen’s (1988) recommendations: approximately 0.01 to 0.09 is considered small, 0.09 to 0.25 is considered medium, and 0.25 and higher is considered large [40].

As the main independent variable, we used T2 sleep quality rather than T1 sleep quality. Had we used T1 sleep quality, that would have suggested that we felt that sleep quality from two years prior (at T1) was influencing financial management behaviors at T3. We suspected that the association between sleep quality and financial management behaviors is not this strong. However, the main disadvantage for making this analytical decision is that it makes the sleep quality and marital satisfaction variables cross-sectional. 

We chose SEM as our analytic estimator for two main reasons. First, SEM models allow multiple regression equations to be estimated simultaneously. Because of this property, we were able to estimate the model for both wives and husbands at the same time and examine actor and partner effects. Second, SEM models estimate a measurement model in addition to estimating the many regression paths (i.e., the structural model). Estimating the measurement model allowed us to obtain findings that accounted for measurement error. That is, we created latent variables for husbands’ and wives’ marital satisfaction and financial management behaviors, thus helping to reduce measurement error [42]. We addressed missing data through the use of the full information maximum likelihood (FIML) method. The variables we used in the SEM had between 0% and 7.7% missing data, with most variables missing less than 5%.

## 3. Results

### Structural Equation Model

**Figure 2:**
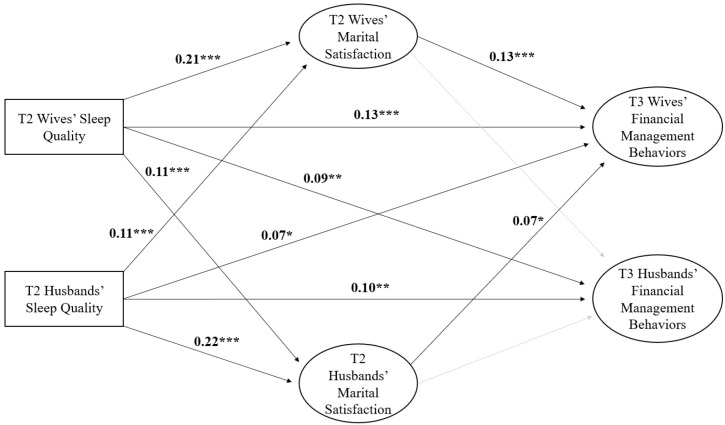
Standardized slope coefficients of the longitudinal actor–partner associations between husbands’ and wives’ sleep quality and financial management behaviors. Note: Rectangles represent observed variables, and ellipses represent latent variables. Gray lines represent statistically insignificant findings. For simplicity, we do not show controlling for husbands’ and wives’ age, education, race, and annual household income. * *p* < 0.05, ** *p* < 0.01, and *** *p* < 0.001.

The fit of the structural equation model to the data was sound. Although the chi-square model fit was statistically significant, this is to be expected given the large sample size. Other fit indices suggested appropriate fit (CFI = 0.99; RMSEA = 0.02; SRMR = 0.02). We discuss the measurement model here only briefly because it was not the focus of our study. The standardized factor loadings for the indicators of the wives’ latent variables are as follows: T3 financial management behaviors loadings ranged from 0.56 to 0.73, and T2 marital satisfaction loadings ranged from 0.88 to 0.90. Respective ranges for the standardized loadings of husbands’ indicators are 0.57 to 0.73 and 0.86 to 0.91. The full results of the measurement model are available from the second author. The direct associations from the SEM are found in Table 2 and in Figure 2.

Wives’ T2 sleep quality was positively associated with their own T2 marital satisfaction (*b* = 0.07. β = 0.21, *p* < 0.001, small effect size) and husbands’ marital satisfaction (*b* = 0.03, β = 0.11, *p* < 0.001, small effect size). Wives’ T2 marital satisfaction was positively associated with their own T3 financial management behaviors (*b* = 0.07, β = 0.13, *p* < 0.001, small effect size). A direct effect between wives’ T2 sleep quality and wives’ T3 financial management behaviors persisted despite having marital satisfaction in the model (*b* = 0.02, β = 0.13, *p* < 0.001, small effect size). Finally, we observed a direct effect between wives’ T2 sleep quality and husbands’ T3 financial management behaviors (*b* = 0.02, β = 0.09, *p* < 0.01, small effect size).

Husbands’ T2 reports of sleep quality were positively associated with their own T2 marital satisfaction (*b* = 0.07, β = 0.22, *p* < 0.001, small effect size) and wives’ marital satisfaction (*b* = 0.03, β = 0.11, *p* < 0.001, small effect size). Husbands’ T2 sleep quality also had direct effects with their own T3 financial management behaviors (*b* = 0.02, β = 0.10, *p* < 0.01, small effect size) and wives’ T3 financial management behaviors (*b* = 0.01, β = 0.07, *p* < 0.05, very small effect size). Neither wives’ T2 marital satisfaction nor husbands’ T2 marital satisfaction was associated with husbands’ T3 financial management behaviors. However, husbands’ T2 marital satisfaction predicted wives’ T3 financial management behaviors (*b* = 0.01, β = 0.07, *p* < 0.05, very small effect size).

As seen in Table 3, two indirect pathways were statistically significant. First, wives’ T2 sleep quality predicted wives’ T3 financial management behaviors through wives’ T2 marital satisfaction (*b* = 0.004, β = 0.03, *p* < 0.05, small effect size), and the 95% confidence interval of the standardized indirect effect did not include zero [0.01, 0.05], suggesting that the indirect effect is statistically different from zero. Similarly, husbands’ T2 sleep quality predicted wives’ T3 financial management behaviors through wives’ T2 marital satisfaction (*b* = 0.002, β = 0.01, *p* < 0.05, small effect size), and the 95% confidence interval did not include zero [0.004, 0.03]. 

Collectively, although the effect sizes are small, we found evidence that both husbands’ and wives’ T2 sleep quality simultaneously predict husbands’ and wives’ T3 financial management behaviors. However, only wives’ T2 marital satisfaction helped explain these actor–partner associations between husbands’ and wives’ T2 sleep quality and wives’ T3 financial management behaviors. Taken together, our model explained 33% of the variance in husbands’ T3 financial management behaviors (large effect size) and 28% of the variance in wives’ T3 financial management behaviors (large effect size), further implicating the importance of sleep quality as a longitudinal predictor of financial management behaviors in newlywed couples.

## 4. Discussion

Building on two previous cross-sectional studies regarding sleep quality and finances [10,11], we examined longitudinal, actor–partner connections between sleep quality and financial management behaviors in newlywed couples. Financial stress in the U.S. has been widespread during the COVID-19 pandemic [1,2], and this might be especially evident for newlywed couples, who often struggle with finances [6,7,8]. Indeed, over 80% Americans in the age range of 18–43, which many newlywed couples might fall under [9], report being stressed about finances [2]. As scholars suggest, financial management behaviors might help mitigate financial stress [3], even under times of widespread economic hardship [4]. Therefore, given the current economic hardship [1,2], understanding additional predictors of financial management behaviors is crucial. 

We found support for our first hypothesis (i.e., that sleep quality will predict financial management behaviors over time for both husbands and wives). That is, we found that both husbands’ and wives’ sleep quality longitudinally predicted their own financial management behaviors, though the effect sizes were small. In line with other scholars’ theorizing [10], we suspect that conscientiousness about sleep longitudinally predicted conscientiousness about finances. However, our hypothesis about marital satisfaction as a mediating variable between sleep quality and sound financial management behaviors was only somewhat supported by our findings. The only indirect pathways that were statistically significant were from husbands’ and wives’ sleep quality through wives’ marital satisfaction to wives’ financial management behaviors. The effect sizes were, again, small. In support of an interdependent perspective of couples’ sleep quality [30], it appears that husbands’ sleep quality might also be somewhat salient in longitudinally predicting wives’ financial management behaviors through wives’ marital satisfaction. We used additional post hoc analyses to test the robustness of our assertion of mediational pathways. We found that marital satisfaction reduced the association between sleep quality and financial management behaviors by 14–21%. Thus, it seems that we do have a partial mediation effect in our analysis.

Additionally, both husbands’ and wives’ marital satisfaction predicted wives’ financial management behaviors with small effect sizes, but neither husbands’ nor wives’ marital satisfaction predicted husbands’ financial management behaviors. On the one hand, these findings are in contrast to Dew et al.’s finding that only husbands’, but not wives’, marital satisfaction was longitudinally associated with their own financial management behaviors [23]. On the other hand, our findings line up with the other literature. For example, previous scholars have found wives’ financial management to be longitudinally connected to marital quality and stability, but husbands’ financial management was not [43], and this lines up with only wives’ marital satisfaction longitudinally predicting their own financial management behaviors. 

Especially because our findings both do [43] and do not [23] align with those of the previous literature, future longitudinal research examining associations between marital processes (e.g., marital satisfaction) and financial processes (e.g., financial management behaviors) is warranted—especially using longitudinal data and methods to disentangle the directionality between these variables [24]. In essence, it appears that husbands’ and wives’ marital satisfaction might longitudinally motivate [23] wives’ conscientious financial management behaviors. Furthermore, future research might consider other potential mechanisms that might link sleep quality and financial management behaviors such as emotion regulation [16,18]. 

Our third hypothesis regarding actor–partner effects received little support, yet a few unexpected actor–partner effects emerged. In addition to their own sleep quality longitudinally predicting their own financial management behaviors, husbands’ sleep quality longitudinally predicted wives’ financial management behaviors with a very small effect size, and wives’ sleep quality longitudinally precited husbands’ financial management behaviors with a small effect size. We suspected the possibility of partner effects, but we expected marital satisfaction to play a mediating role, which only held true for one indirect partner effect. We suspect that the direct partner effects could be explained by a contagious conscientiousness between partners. As one partner sees and experiences his/her partner sleeping well, perhaps his/her conscientiousness about health behaviors such as sleep might motivate his/her own conscientiousness with finances [10].

In all, it appears that husbands’ and wives’ sleep quality is longitudinally associated with husbands’ and wives’ financial management behaviors. Nonetheless, we recognize that the effect sizes of our longitudinal findings are small. As such, we do not recommend sleep quality as the only—or even the main—aspect for financial practitioners to consider for newlywed couples struggling with their financial management. However, because we found that husbands’ and wives’ sleep quality predicted their own and their partner’s financial management behaviors following a one-year time span between measurement periods, we maintain that the small effect sizes of the longitudinal associations between sleep quality and financial management behaviors have practical importance for practitioners who help newlywed couples. 

### 4.1. Implications for Practitioners

Because empirical examination of sleep quality predicting financial management behaviors is still in its infancy, we suspect that many financial practitioners (i.e., financial planners, counselors, educators, and clinicians) who help newlywed clients with their financial management might not have considered sleep quality in their interventions. However, we submit that sleep quality might be considered as part of a multifaceted financial behavior intervention for newlywed couples [10]. For example, to help clients improve financial management behaviors, financial practitioners might help clients regulate their emotions [18], improve their marital satisfaction [23], improve financial capability, and lessen financial stressors [44]. In multifaceted interventions such as these, financial practitioners might also consider helping newlywed clients improve their sleep quality. Specifically, sleep experts recommend that individuals ages 18–60 get at least seven hours of sleep per night [45]. As such, financial practitioners might suggest that their newlywed clients ages 18–60 get at least seven hours of sleep at night to improve their financial management behaviors. 

In explaining connections between sleep quality and financial management behaviors, we suggest explaining these connections through the lens of the FFMP [13]—especially conscientiousness. That is, financial practitioners might describe sleep quality as representing conscientiousness about health behaviors that might motivate conscientiousness with finances [10]. To make these connections more concrete for newlywed clients, describing how quality sleep might promote better emotion regulation [16] and better emotion regulation might be connected to better financial management behaviors [18] might be useful for clients. Additionally, in line with the sleep-in-marriage theory [19], practitioners might consider the dyadic nature of newlywed couples’ sleep. That is, our findings suggest that helping both newlywed husbands and wives improve their sleep quality might help both husbands and wives improve their financial management behaviors over time. Additionally, although we found limited evidence of marital satisfaction as a mediating variable, husbands’ and wives’ sleep quality might also be connected to better marital satisfaction for wives, which might motivate wives’ financial management behaviors. 

In support of Dew et al. [23], we found some evidence that husbands’ and wives’ marital satisfaction might motivate wives’ financial management behaviors. Although many researchers have the direction going from financial management behaviors to marital satisfaction [24], we posit that marital satisfaction as a motivator for financial management behaviors also makes theoretical sense. For example, it might be unrealistic to suggest that couples in a strained relationship should manage their finances better to improve their marital satisfaction [25]. That is, these couples might first need to develop their marriage, and then positive financial management behaviors might follow. Therefore, based on our findings and other preliminary evidence [23], we cautiously recommend marital satisfaction as an intervention for improving newlywed wives’ financial management behaviors, while also recognizing that future research is needed. Financial practitioners might consider referring newlywed couples to a financial therapist to help the couple develop their marriage to help develop their financial management behaviors.

### 4.2. Limitations

Like most social science research, this study had limitations. First, although we used two waves of longitudinal data and accounted for relevant control variables, we acknowledge that our findings are not causal. A randomized control group experimental design can establish causality, but we did not employ such a design. However, as a robustness check, we assessed the longitudinal effects of T2 financial management behaviors predicting T3 sleep quality (i.e., the reverse of the direction we tested in this study). We found no direct associations between T2 financial management behaviors and participants’ T3 sleep quality. This contrasts with the direct actor–partner associations we found from T2 sleep quality to T3 financial management behaviors. One indirect association did arise in these findings. Wives’ T2 financial management behaviors were positively associated with wives’ T3 sleep quality through wives’ T2 marital satisfaction. We found a similar indirect effect in our first model (i.e., wives’ T2 sleep quality to wives’ T2 marital satisfaction to wives’ T3 financial management behaviors). For this particular indirect effect, then, we cannot be sure about the direction of the effect. However, given that the direct actor–partner associations existed between T2 sleep quality and T3 financial management behaviors, our findings suggest that sleep quality predicts financial management more than the other way around.

A second limitation is that our sample had some generalizability issues. Because our analytical sample only included newlywed couples, our findings only apply to that population. Additionally, we included only different-sex couples from the CREATE data set due to the small sample size of same-sex couples in the data. As such, our findings might only apply to different-sex newlywed couples. Finally, our data also contained a weakness that often plagues surveys—sampling bias. Even though CREATE used stratified random sampling, it may be that couples who were happier in their marriage were also more likely to participate in CREATE relative to unhappier newlywed couples. Furthermore, we used data only from couples who stayed married through T3. Thus, our sample may represent findings from happier, more stable newlywed couples.

## 5. Conclusions

Within the context of its limitations, and also in spite of them, this study adds to the literature on sleep quality, marital satisfaction, and financial management behaviors in newlywed couples. As newlywed husbands and wives are intentional about getting quality sleep, both husbands’ and wives’ financial management behaviors over time might benefit. For wives’ financial management behaviors, these connections may be indirect through wives’ marital satisfaction. In essence, financial practitioners who help newlywed couples might consider employing practices in line with these longitudinal associations. In short, we suggest that, for newlywed couples, both partners’ bedtime may be longitudinally connected to both partners’ management of their budget. 

## Figures and Tables

**Table 1:** Demographic statistics.

Demographic	M or %	SD	Min–Max
Husbands’ Age at T1	29.75	5.56	16–63
Wives’ Age at T1	27.79	4.83	17–63
Husbands’ Report of Annual Household Income at T2	8.04	4.15	1–16
Wives’ Report of Annual Household Income at T2	7.90	4.17	1–16
Husbands’ Highest Education Obtained at T1	3.79	1.51	1–7
Wives’ Highest Education Obtained at T1	4.13	1.44	1–7
Husbands Who Identified as Black ^a^	9.4%	--	0–1
Husbands Who Identified as Hispanic ^a^	11.8%	--	0–1
Husbands Who Identified as a Race/Ethnicity other than White, Black, and Hispanic ^a^	11.4%	--	0–1
Wives Who Identified as Black ^a^	7.4%	--	0–1
Wives Who Identified as Hispanic ^a^	12.2%	--	0–1
Wives Who Identified as a Race/Ethnicity other than White, Black, and Hispanic ^a^	13.7%	--	0–1

Note: ^a^ Reference group is those who identified as White. T1 represents wave one. An annual household income score of 8 suggests an annual household income between $70,000 and 79,999. A highest-education-level-obtained score of 4 suggests the completion of an associate degree.

**Table 2:** Direct associations between husbands’ and wives’ sleep quality, marital satisfaction, and financial management behaviors.

Construct	W T2 Marital Satisfaction			H T2 Marital Satisfaction			W T3 Financial Management Behaviors			H T3 Financial Management Behaviors		
	*b*	SE_b_	β	*b*	SE_b_	β	*b*	SE_b_	β	*b*	SE_b_	β
W T2 Sleep Quality	0.07 ***	0.01	0.21	0.03 ***	0.01	0.11	0.02 ***	0.005	0.13	0.02 **	0.005	0.09
H T2 Sleep Quality	0.03 ***	0.01	0.11	0.07 ***	0.01	0.22	0.01 *	0.005	0.07	0.02 **	0.005	0.10
W T2 Marital Satisfaction	--	--	--	--	--	--	0.07 ***	0.02	0.13	0.03	0.02	0.05
H T2 Marital Satisfaction	--	--	--	--	--	--	0.01 *	0.005	0.07	0.04	0.03	0.08
*R* ^2^	0.08			0.07			0.28			0.33		

Note: H represents husbands’, and W represents wives’. Control variables of age, income, race/ethnicity, and education are not shown for the sake of clarity. * *p* < 0.05, ** *p* < 0.01, and *** *p* < 0.001.

**Table 3:** Summary of the statistically significant direct and indirect associations between husbands’ and wives’ sleep quality and financial management behaviors.

Pathways	*b*	SE_b_	β; [95% CI]
W T2 Sleep Quality → W T3 Financial Management Behaviors	0.02 ***	0.005	0.13 [0.07, 0.19]
W T2 Sleep Quality → H T3 Financial Management Behaviors	0.02 **	0.005	0.09 [0.03, 0.15]
H T2 Sleep Quality → H T3 Financial Management Behaviors	0.02 **	0.005	0.10 [0.04, 0.16]
H T2 Sleep Quality → W T3 Financial Management Behaviors	0.01 *	0.005	0.07 [0.01, 0.13]
W T2 Sleep Quality → W T2 Marital Satisfaction → W T3 Financial Management Behaviors	0.004 *	0.002	0.03 [0.01, 0.05]
H T2 Sleep Quality → W T2 Marital Satisfaction → W T3 Financial Management Behaviors	0.002 *	0.001	0.01 [0.004, 0.03]

Note: H represents husbands’, and W represents wives’. CI stands for confidence interval; * *p* < 0.05, ** *p* < 0.01, and *** *p* < 0.001.

## Data Availability

The data used in the current study are not publicly available because participants have not provided such consent. However, the analysis code/syntax used in the current study is available upon request from the second author.
